# Relationship Between Sociosexuality and Condom Use Frequency Among Young French College Students

**DOI:** 10.5964/ejop.6793

**Published:** 2023-02-28

**Authors:** Kamel Gana, Diana Arshakyan

**Affiliations:** 1 Department of Psychology, University of Bordeaux, Bordeaux, France; Glasgow Caledonian University, Glasgow, United Kingdom

**Keywords:** sociosexuality, condom use, college students, validation, SOI-R, French

## Abstract

Sociosexuality, conceptualized as individual differences in attitudes, behaviors, and desires for casual sex, is reflected in “hookup culture” where risky sexual behaviors should not be overlooked. The main objectives of this study were (a) to provide a first French adaptation of the SOI-R and to evaluate its psychometric properties, and (b) to examine the relationship between sociosexuality and condom use among young college students (N = 1037, mean age = 18.7 years, SD = 1 year). A path model hypothesizing links between dispositional optimism, boredom proneness, sexual orientation, age, gender (as correlated exogenous/independent variables), sociosexuality (as mediation variable), and condom use (as output variable), was specified and tested. Findings showed gender and sexual orientation differences in sociosexuality. As expected, males as well as non-heterosexual individuals endorsed more sociosexuality than the others. Optimism, but not boredom, predicted a higher level of sociosexuality. Sociosexuality positively predicted safer sex. Sociosexual orientation was not associated with condomless sex. It would seem that sexual freedom does not necessarily mean irresponsible sexual adventures for the young college students in our study.

Adolescents and young adults are particulary at risk for sexually transmitted infections (STIs; Olmstead et al., 2015). Emerging adulthood is a period of experimentations and possibilities (Arnett, 2016; Morgan & van Dulmen, 2021). Sexual experiences are inherent to the socialization process celebrating autonomy and independence, so much desired by these emerging adults (Arnett, 2016). The increased sexual permissiveness in the Western culture gives young people freedom and autonomy to explore and experiment with dating and sexual relationships (Claxton & van Dulmen, 2013). Today, emerging adults in the Western culture believe it is necessary to explore the different possibilities available to them (Arnett, 2016). Online dating applications (i.e., “hookup apps”, such as Tinder; see LeFebvre, 2018; Sevi, 2019a) seem to facilitate or even incite and encourage these experiences and possibilities (Sumter et al., 2017). In such a context, condom use is one of the most effective ways to prevent STIs.

Despite multiple prevention campaigns implemented by public health authorities (e.g., prevention website www.onsexprime.fr), developed for teens by Public Health France, a significant number of adolescents and young college students admit to engaging in risky sexual behaviors (e.g., unprotected sex). For instance, according to 2014 World Health Organization statistics (WHO, 2014), in France, the percentage of young people (15 years old) who used a condom in intercourse was 65% in women and 79% in men. According to Régnier-Loilier (2020), 24% of French students aged 17 or older did not use a condom at first intercourse. These observations are all the more serious as young people are currently immersed in “hookup culture” which is facilitated by online applications (Aubrey & Smith, 2016). Although the precise definition varies between studies (Lewis et al., 2013), hooking up is broadly defined as an uncommitted sexual encounter, resulting in sexual activities, which can range from kissing to oral sex or vaginal and/or anal intercourse that occurs between individuals who are not in a current dating relationship (Garcia et al., 2019). Hookups occur at high rates on university and college campuses (Dai et al., 2018), ranging from 58% (Kalish & Kimmel, 2011; Kuperberg & Padgett, 2015) to 85% (Lambert et al., 2003).

Various factors could explain hooking up behaviors (Fielder et al., 2013; Owen et al., 2010). Sociosexuality is one of them (Botnen et al., 2018; Sevi et al., 2018). According to Gangestad and Simpson (1990), sociosexuality refers to differences in individuals’ implicit prerequisites to entering a sexual relationship. According to Penke and Asendorpf (2008), it refers to individual differences in attitudes, behaviors, and desires for casual sex. These individual differences represent a continuum that ranges from the unrestricted sociosexual orientation to the restricted sociosexual orientation. The former orientation is characterized by the predisposition and willingness to engage more easily and more often in uncommitted sexual relationships. The latter orientation is characterized by the need to have more time, stronger attachment, commitment, and closeness with romantic partners before willing to engage in sexual intercourse with them. Sevi (2019b) found that Tinder use for short-term mating, but not for long-term mating, was correlated to sociosexuality. In addition, Tinder users with higher scores on sociosexuality significantly showed greater motivation to use Tinder for short-term mating. Moran et al. (2018) found that sociosexuality was related to hook up behavior in the use of the Snapchat dating app. Indeed, their results showed that unrestricted individuals more frequently use Snapchat to gain sexual access, ask for a hookup, and to continue sending naked Snapchats. In the same vein, Botnen et al. (2018) found that independent of gender, unrestricted sociosexuality predicted the use of dating apps.

To evaluate sociosexuality, Simpson and Gangestad (1991) developed and validated the Sociosexual Orientation Inventory (SOI) comprising 7 items. Penke and Asendorpf (2008) developed and validated the Revised Sociosexual Orientation Inventory (SOI-R). It is a 9-item self-report questionnaire rated on a 9-point Likert scale. It assesses overall sociosexual orientation including three dimensions: (1) sociosexual behavior, (2) attitudes toward sex without commitment, and (3) sociosexual desire. Scores range from 9 to 81 with higher scores indicating more unrestricted sociosexuality. The SOI-R has been used in many languages such as Hungarian (Meskó et al., 2014), Japanese (Nakamine & Komura, 2016), Portuguese (Neto, 2016), and Spanish (Barrada et al., 2018).

Research on determinants of condom use remains more necessary than ever within a “hookup culture”. For instance, Ballester-Arnal et al. (2017) found that negative affectivity (e.g., depressed mood, boredom) acted as risk factors leading to condomless sex, while self-efficacy acted as a protective factor against unsafe sex. Also, sexual sensation seeking was found to be associated with more inconsistent condom use as well as a higher number of sexual partners (Davis et al., 2014). Carvajal et al. (1998) found that dispositional optimism (i.e., high expectancies for positive outcomes in the future and low expectancies for negative events in the future) was a protective factor regarding adolescents’ intentions to avoid engaging in condomless sex. Contrary to negative affectivity, positive psychological resources (e.g., hope, optimism, self-esteem) seem to promote, directly or indirectly, safer sex (Broaddus & Bryan, 2008). However, to our knowledge, the link between condom use and sociosexual orientation have not been published so far. The present study aimed to fill this gap.

## Objectives of the Study

The aim of this study was twofold:

First, the study was designed to investigate the psychometric properties (i.e., factor structure, gender measurement invariance, reliability) of the first French adaptation of the Revised Sociosexual Orientation Inventory (SOI-R). There is no measure of sociosexuality in French, and the SOI-R has never been adapted to the French population.

**Figure 1:**
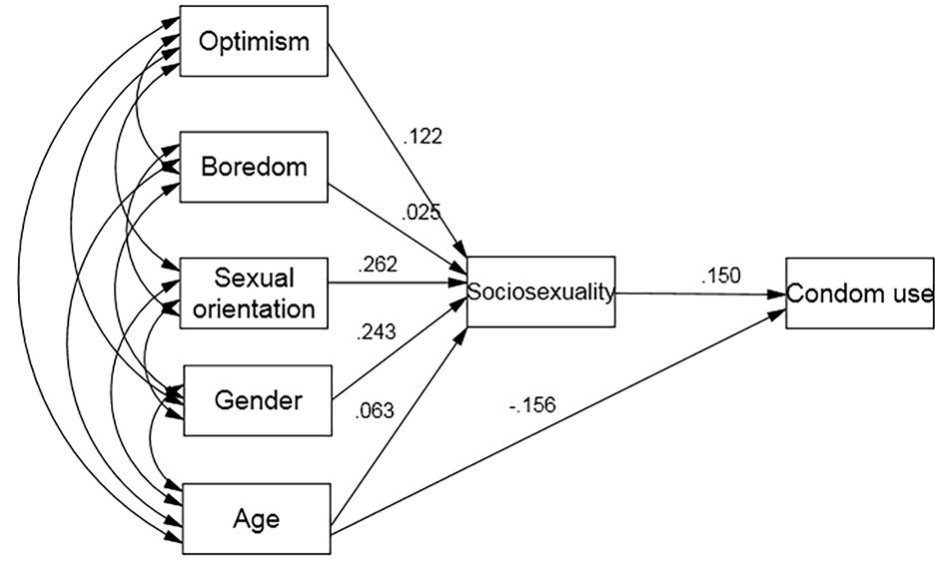
Path Diagram of the Hypothesized Model Illustrating Direct and Indirect Effects of Optimism, Boredom, Sexual Orientation, Gender, Age (Correlated Exogenous Variables), and Sociosexuality (Mediation Variable) on Condom Use (Output Variable) *Notes*. The direct path from age to condom use was added as suggested by the modification indices. Standardized coefficients from the path analysis are displayed. Only values .063 and .025 are not statistically significant. Gender coded as F = 1, M = 2; Sexual orientation coded as Heterosexual = 1, Non-heterosexual = 2.

Second, the study was designed to explore and investigate relationships between sociosexuality, dispositional optimism, boredom proneness, and safer sex (i.e., condom use frequency). Specifically, we translated these relationships into a path model. A diagrammatic representation of this model is depicted in Figure 1, which provides the hypothesized relationships among our variables. This model expressed the following a priori specifications: dispositional optimism and boredom proneness (as personality traits), as well as sexual orientation, should act as predictors of sociosexuality, which, in turn, should impact condom use (as a behavior). Thus, sociosexuality should act as a mediator variable between personality traits and ultimate behavior (i.e., condom use). More precisely: (1) optimism and boredom should positively predict sociosexuality, meaning the higher the optimism or boredom the more sociosexuality is unrestricted. Our rationale for these predictions was that bored people may be disinterested in long-term relationships given the potential lack of stimulation and monotony that may exist in stable relationships, and that optimism may bias people toward favoring hookup lifestyle (Carvajal et al., 1998); Indeed, optimism is a positive psychological resource related to self-esteem (Andersson, 2012a; Caprara et al., 2013), hope, self-efficacy (Carver & Scheier, 2014), extraversion (Furnham & Cheng, 2018), goal engagement and perceived success (Heckhausen & Wrosch, 2016), and greater social network size and social resources (Andersson, 2012b). Paraphrasing Carver and Scheier (2014), we can say that someone can be optimistic because he/she has great confidence in his/her abilities or because he/she believes other people like and look out for him/her; (2) unrestricted sociosexual orientation should positively predict safer sex, which is the *sine qua non* condition to preserve the sexual lifestyle; for instance, Cooper and Orcutt (2000) showed that youth engaging in casual sex tend to use condoms more often; and (3) because there are gender differences as well as sexual orientation differences in sociosexuality, these variables should have indirect effects on condom use, via sociosexuality. Gender differences in sociosexuality demonstrated cross-cultural universality (Schmitt, 2005). For instance, using data from 53 nations and from over 200,000 participants, Lippa (2009) found that sociosexuality showed consistent sex differences across nations. Age was found to be related to the frequency of sexual activity among adolescents and young adults (Herbenick et al., 2010). Even if the age range at colleges is rather low, a signle additional year at the college can mean a lot of cumulated experience and cultivation.

## Method

### Participants and Procedure

A total of 1037 college students, recruited in 2019 via social media platforms, participated in this study. There were 802 women, 231 men, and 4 did not specify their gender and were then deleted in some statistical analyses (e.g., gender differences). They were between 17 and 20 years old (*M* = 18.7 years, *SD* =1 year). Because the median age of first sexual intercourse is estimated to be around 17 and a half years for young people in France (Maillochon et al., 2016), we set the minimum age for participation in our study at 17 years old. Seventy-seven percent (77%) self-identify as heterosexual, 15% as bisexual, 2.5% as homosexual, 4.5% as pansexual, and 0.5% responded “other”.^1^1Because only seven participants answered “other”, they were dropped from the analyses dealing with sexual orientation. They filled out individually the questionnaire online via Google Form. All participants were invited to sign an informed consent. Also, they were informed of the voluntary and anonymous nature of the study, of their right to not answer any question that made them uncomfortable as well as their right to withdraw from the study at any time. Minor participants were asked to inform their parents before filling out the questionnaire. The institutional academic team approved our research protocol.

### Measures

The SOI-R (Penke & Asendorpf, 2008) is a 9-item self-report questionnaire rated on a 9-point Likert scale (e.g., ‘‘With how many different partners have you had sex within the past 12 months?’’, ‘‘Sex without love is ok’’). Following the ITC Guidelines for Translating and Adapting Tests (International Test Commission, 2017), we convened a committee of psychologists to examine, discuss, review, and adapt three French translations of the SOI-R in order to propose a single consensual version. A pilot study with a small sample (*n* = 30) was carried out to evaluate the clarity of the items of the French adapted version. No difficulty in understanding the items or the scale instruction was noted.

The Life Orientation Test-Revised (LOT-R) is a self-report measure of optimism developed by Scheier et al. (1994), and adapted and validated for French by Trottier et al. (2008). The LOT-R is a 6-item scale (with four filler items) that evaluates respondents’ generalized expectations of positive and negative outcomes (e.g., “Overall, I expect more good things to happen to me than bad”). Higher scores indicate higher levels of optimism. Cronbach’s α was .79.

The Boredom Proneness Scale (BPS) is a self-report measure of boredom proneness, developed by Farmer and Sundberg (1986). A short version validated by Gana et al. (2019) was used. It comprises 8 true-false items in which participants are asked to respond by answering yes or no about how they felt in general (e.g., “Many things I have to do are repetitive and monotonous”). Higher scores indicate higher levels of boredom. Cronbach’s α was .67.

Condom use frequency was assessed by a single item asking the respondents to indicate how often they use a (male or female) condom when they have sex on a 6-point frequency scale ranging from 0 “not concerned”, 1 “never” to 5 “every time”. Because our study focused on young people, some of whom are sexually inactive (never engaging in sexual activity), the response option "not concerned" was accompanied by clarifications (“abstinence, sexless relationship”). According to Fonner et al. (2014), to control for abstinence, those who answered “not concerned” (*n* = 209) were excluded in some statistical analyses.

Sexual orientation was assessed with one item that asked youths to self-identify, using five options: 1 = exclusively heterosexual, 2 = bisexual, 3 = exclusively homosexual, 4 = pansexual, and 5 = other.

### Data Analyses

Data analyses proceeded in three steps. First, we performed confirmatory factor analyses to test the structural validity of the SOI-R. Three models were specified and tested: (a) a single-factor model that assumes that sociosexuality is unidimensional, (b) a three-factor oblique model (i.e., behavior, attitude, and desire), and (c) a bifactor model that includes a general factor “g”, in which all the items load, as well as the three specific factors underlying the SOI-R (i.e., behavior, attitude, and desire) on which respective items load (Gana & Broc, 2019). Second, we tested gender invariance of the SOI-R. Third, we examined descriptive statistics for scale sum scores (e.g., correlations, gender differences). Finally, a path analysis was performed to test our specified model (Figure 1). All our statistical analyses were performed using using lavaan and semTools packages within R software (R Project for Statistical Computing).

## Results

### Analysis of the French Version of the SOI-R

#### Factor Structure of the French Version of the SOI-R

The competing measurement models underlying the SOI-R were fitted by using the maximum likelihood estimation with robust standard errors (MLR) because the multivariate normality was affected (Mardia’s coefficient = 32.29, *p* < .001).

Table 1 summarizes the results for the three competing measurement models of the SOI-R. The χ2 test does not support the fit of any of these models. However, because this statistic is sensitive to sample size (Gana & Broc, 2019), alternative fit indices are used. The worst model is the single-factor model. The three-factor oblique model of the SOI-R fitted slightly better our data than the bifactor model. Thus, the three-factor model provided a good conceptualization of SOI-R items’ structure. And because the 3-factor model is consistent with the extant SOI-R literature, the present results would help extend the literature.

**Table 1:** Goodness-of-Fit Indices of the Competing Measurement Models of the SOI-R, Using MLR Estimation Method (N = 1037)

Models	χ^2^	*df*	*p-*value (χ^2^)	CFI	TLI	RMSEA
1-factor model	2054.52	27	.000	.576	.435	.269
3-factor model	130.12	24	.000	.980	.970	.065
Bifactor model	112.30	18	.000	.983	.966	.071

*Note*. *df* = degrees of freedom; CFI = comparative fit index; TLI = Tucker-Lewis Index; RMSEA = root mean square error of approximation.

Examination of factor loadings from the three-factor model shows that all the items loaded significantly on their respective factors, and they yielded coefficients of .70 or higher, min-max = .718 [item9] to .978 [item3]. The correlations between the three factors were moderate, ranged from .339 (Behavior with Desire) to .546 (Attitude with Desire).

#### Gender Measurement Invariance

Prior to performing MG-CFA, we tested the three-factor model separetly in each gender group. Table 2 shows the goodness-of-fit indices indicating that both models fit very well the data. Thus, the equivalence of this model was tested across gender by imposing a series of increasingly stringent constraints between groups, i.e., configural [no equality], weak [equal loadings], strong [equal loadings and intercepts] and strict invariance [equal loadings, intercepts, and error variances]. As shown in Table 2, model comparisons indicated that the factor loadings can be assumed to be equal across gender (i.e., weak invariance), since ΔCFI and ΔRMSEA are below the proposed cut-point of 0.01 (Chen, 2007). Also, strict and strong invariance models were tenable, allowing gender comparisons of the the SOI-R scores.

**Table 2:** Goodness-of-Fit Indices of Gender Measurement Invariance Models of the SOI-R, Using MLR Estimation Method

Models	χ^2^	*df*	*p* (χ^2^)	CFI	∆CFI	TLI	RMSEA	∆RMSEA
CFA								
Female	95.62	24	< .001	.982	-	.973	.063	-
Male	53.68	24	< .001	.976	-	.963	.074	-
MG-CFA								
Configural	150.38	48	< .001	.981	-	.971	.066	-
Weak	183.63	54	< .001	.975	.006	.967	.070	.004
Strong	216.57	60	< .001	.970	.005	.964	.073	.003
Strict	252.89	69	< .001	.960	.010	.959	.078	.005

*Note*. *df* = degrees of freedom; CFI = comparative fit index; TLI = Tucker-Lewis Index; RMSEA = root mean square error of approximation.

#### Reliability of the SOI-R scores

The coefficients α (Cronbach, 1951) and ω (Raykov, 2001) values were .91, 95% CI [.90, .92] and .92, 95% CI [.91, .93] respectively for the behavior subscale, .85, 95% CI [.83, .86] and .85, 95% CI [.84, .86] respectively for the attitude subscale, .84, 95% CI [.82, .86] and .85, 95% CI [.84, .87] respectively for the desire subscale, and .86, 95% CI [.85, .87] and .93, 95% CI [.92, .94] respectively for the total score. The deletion of any item was not likely to improve the reliability of the scale.

### Descriptive Statistics

Zero-order correlations among study variables are shown in Table 3. Sociosexual orientation is significantly and positively correlated with dispositional optimism, but not with boredom proneness.

**Table 3:** Intercorrelations of the Variables Used in This Study (N = 1033, Except Correlations With Condom Use N = 826)

Variables	1	2	3	4	5	6	7
1. Optimism	1.00						
2. Boredom	-.340^a^	1.00					
3. Sexual orientation^b^	-.107^a^	.082^a^	1.00				
4. Gender (F = 1, M = 2)	.006	.044	-.010	1.00			
5. Age	.045	-.097^a^	.044	-.093^a^	1.00		
6. Sociosexuality	.083^a^	-.001	.226^a^	.218^a^	.043	1.00	
7. Condom use	.031	-.025	.008	.051	-.148^a^	.143^a^	1.00

^a^ Significant at at least *p* < .05. ^b^ Sexual orientation was coded as Heterosexual = 1 and Non-heterosexual = 2.

Gender differences in sociosexuality revealed that males (*M* = 36.23, *SD* = 13.92) endorsed more unrestricted sociosexuality than females (*M* = 28.78, *SD* = 12.31), *t*(1031) = 7.89, *p* < .001, *d* = .57. ANOVA yielded significant sexual orientation differences in sociosexuality: *F*(3,1026) = 18.94, *p* = .000, η^2^ = .052. Post-hoc comparaisons revealed that, (a) bisexuals (*M* = 36.20, *SD* = 13.34), homosexuals (*M* = 37.23, *SD* = 18.71) and pansexuals (*M* = 34.04, *SD* = 11.11) were not statistically different from one another, and (b) they endorsed more unrestricted sociosexuality than heterosexuals (*M* = 28.80, *SD* = 12.46). Thus, for subsequent analyses, we created two groups: heterosexual participants (*n* = 799) and non-heterosexual participants (i.e., homosexuals, bisexuals, pansexuals; *n* = 238). Heterosexual participants displayed less unrestricted sociosexual orientation (*M* = 28.80, *SD* = 12.46) than non-heterosexual participants (*M* = 35.80, *SD* = 13.53), *t*(1035) = 7.45, *p* < .001, *d* = .53.

Among females, heterosexuals (*M* = 26.96, *SD* = 11.86) and non-heterosexuals (*M* = 34.67, *SD* = 11.88) differed in sociosexuality, *t*(800) = 7.81, *p* < .001, *d* = .59. The same is true for males (heterosexuals: *M* = 34.87, *SD* = 12.52; non-heterosexuals: *M* = 41.84, *SD* = 17.37), *t*(229) = 3.07, *p* < .005, *d* = .53.

### Path Analysis

To evaluate the plausibility of the hypothetical model presented in Figure 1, a path analysis was performed using the maximum likelihood method of estimation. Because the ultimate endogenous variable in this model was condom use frequency, participants who answered that they were not concerned by this issue (*n* = 209) were excluded, leaving 826 cases for this analysis. The goodness-of-fit results indicated a poor model fit (CFI = .882, TLI = .740, and RMSEA = .065). Using the modification indices, the model was modified to include a direct effect (path) of age on condom use. Thus, the respecified model provided an excellent fit to the sample data (χ^2^ = 1.79, *df* = 4, ns; CFI = 1.00, TLI = 1.00, RMSEA = .000). Dispositional optimism (β = 122, *p* < .001) but not boredom proneness (β = .025, ns) proved to have a significantly positive effect on sociosexual orientation. Gender and sexual orientation showed significant positive effects on sociosexuality (β = .243 and β = .262 respectively). Age showed a negative direct effect on condom use (β = -156, *p* < .001). Sociosexuality (β = .150, *p* < .001) showed a significant positive effect on condom use.

Indirect effect analyses revealed that the indirect positive effects of optimism and sexual orientation on condom use were statistically significant (*p* < .05). These effects were completely mediated by sociosexuality (because they had no significant direct effects on condom use).

## Discussion

The current study is the first to evaluate the psychometric properties of the French version of the SOI-R. Overall, the results support the psychometric properties of this scale in a French sample. Our measures of reliability indicated an adequate level of internal reliability for the SOI-R scores. These results are comparable with those obtained by Penke and Asendorpf (2008). Concerning structural validity, our findings add further evidence to the body of studies confirming the three-factorial structure of the SOI-R. Indeed, Nascimento et al. (2018) confirmed the existence of these three related factors in a Brazilian sample. Barrada et al. (2018) evaluated the psychometric properties of the SOI-R in a Spanish sample. Their results support the tridimensional structure in this population. The Portuguese version of the SOI-R validated by Neto (2016) yielded the three-factor structure. In addition, our findings suggest that the strict invariant three-factor model of the SOI-R fit the data fairly well for women and men. This means that, (a) all important parameters of the measurement model were found to be equivalent across gender, (b) the items in the French version of the SOI-R were not prone to any gender bias, and (c) gender differences in the means, variances, and covariances of the items are entirely attributable to gender differences in the latent common factors (Millsap & Cham, 2012). Thus, one can conclude that any differences between gender can be interpreted as reflecting actual differences in sociosexual orientation rather than differences arising from measurement bias. Thus, as for the Spanish version (Barrada et al., 2018), scores in the French version of the SOI-R are gender-free.

Concerning the relationships between dispositional optimism, boredom proneness, sociosexuality, gender, sexual orientation, and condom use, our main findings are as follows:

First, as expected males reported more unrestricted sociosexuality than females. This finding is in line with those observed in various Western and non-Western societies (Lippa, 2009; Nascimento et al., 2018; Neto, 2016; Schmitt, 2005, 2007; Zheng et al., 2014). Few theroretical models can help understanding gender differences in sociosexuality: parental investment theory, sexual strategies theory, and social learning theory.

According to parental investment theory (Trivers, 1972) and sexual strategies theory (Buss & Schmitt, 1993), men should possess more unrestricted sociosexual orientation than women across human cultures (Lippa, 2009; Schmitt, 2005; Sevi et al., 2018). Schmitt (2005) pointed out that gender differences in sociosexuality are culturally universal. Indeed, parental investment theory argues that there are, in general, gender differences in parental care, meaning the investment of time, energy, and resources that each parent devotes to the survival of their offspring. Females are known to provide a considerably higher investment in their offspring than males. This asymmetry governs sexual selection and mating strategies. Thus, since the less invested gender, i.e. males, can abandon parental care to improve reproductive success at a low cost, these individuals will be more disposed to desert their partners for sexual opportunities (Mogilski, 2021). That is, as males, in general, are more invested in mating than parenting, they likely endorse more unrestricted sociosexuality than females do (Schmitt, 2005).

According to social learning theory, women's sexuality is shaped to a greater degree by sociocultural variables such as educational background and religion than is men's (Baumeister, 2000). It is now recognized that women's sexuality is more affected by cultural reproduction and social reproduction than that of men (Agocha et al., 2014). Indeed, the sexual double standard—meaning that adolescent boys are seen to possess an independent, active and irrepressible sex drive, while adolescent girls are believed to be passive receptacles of male sexual interest, having little or no one’s own sexual desire (Caron et al., 2020; Fjær et al., 2015)—is still tirelessly reproduced culturally in each male-dominated society. Koomson and Teye-Kwadjo (2021) found that Ghanaian men seem to view uncommitted sex as an appropriate sexual behavior, conforming to prevailing masculine sexual norms. Consequently, women are more likely to experience negative social, physical, and emotional consequences associated with hooking-up compared to their male counterparts. For instance, results from the prospective study among high school students performed by Dubé et al. (2017) showed that one-night stand relationships increased only girls’ psychological distress and both their alcohol and drug use. According to these authors, regret may increase psychological distress. Thus, in order to avoid these consequences, young girls seem more reluctant than men to engage in hookups.

Second, for both males and females, heterosexual participants reported more restricted sociosexuality than non-heterosexual participants. This finding is also in line with those observed in the literature (Lippa, 2020; Semenyna et al., 2018). For instance, Waldis et al. (2021) found that homosexual men reported more unrestricted sociosexuality than heterosexual men. The same authors (Waldis et al., 2020) found the same result among heterosexual and homosexual women.

Third, of the two personality traits included in our study, only dispositional optimism, but not boredom proneness, showed a positive significant effect on sociosexual orientation. Higher levels of optimism predicted more unrestricted sociosexuality. Optimism is a positive psychological resource related to self-esteem, hope, and self-efficacy (Carver & Scheier, 2014) shaping an individual’s response to challenging situations and negative outcomes, such as failure with online/offline dating. Indeed, optimistic individuals view failure as a temporary disappointment that they can resolve.

However, our hypothesis that boredom could lead to sensation-seeking by multiplying dating opportunities had not been confirmed. For instance, Jonason et al. (2019) found that negative affectivity was positively associated with short-term mating orientation. It is worth noting that the internal consistency reliability of the 8-item self-report measure of boredom (.67) was below the desirable value (greater than .70). Although Cronbach’s alpha is sensitive to the number of items in the scale (McNeish, 2018), using low reliable scale scores could lead to poor statistical results (Hair et al., 2014). Thus, further research, using more reliable tools, is needed here to explore the links between negative affectivity and sociosexuality.

Fourth, unrestricted sociosexual orientation does not seem to be a risk factor for safer sex among our young college students. On the contrary, unrestricted sociosexual orientation predicts condom use and thus may act as a protective factor for risky sexual behaviors. Within the socialization process and dynamic of these young college students, unrestricted sociosexual orientation may be an assumed and responsible choice. Sexual freedom does not necessarily mean risky sexual behaviors. Although this result is gratifying, efforts regarding prevention campaigns against STIs must not be slackened. Do the initial fear and vigilance dissipate with time and sexual experiences among these young college students?

Finally, optimism indirectly predicted safer sex. This result may sound contreintuitive. However, one can prudently assume that optimism as a disposition promotes sociosexuality, but higher sociosexual orientation comes along with higher cautiousness. Nevertheless, there is a lack of research that has investigated the links between positive affectivity and condom use.

The current study is not without limitations. First of all, its cross-sectional design precludes us from making any causal inferences (e.g., maybe from students high in sociosexuality only optimistic people fully participated). Also, given the complexity of psychological functioning and the range of potential indicators that could have been used, our path model is certainly incomplete. Indeed, condom use attitudes, gender role orientation, masculinity ideology (Noar & Morokoff, 2002; Shearer et al., 2005) could have been integrated into our model. Replication and extension of our study would be an interesting approach.
